# Prevalence of and Predictors for Frequent Utilization of Emergency Department: A Population-Based Study

**DOI:** 10.1097/MD.0000000000001205

**Published:** 2015-07-24

**Authors:** Mingchung Ko, Yaling Lee, Chuchieh Chen, Pesus Chou, Dachen Chu

**Affiliations:** From the Department of Emergency Medicine and Surgery, Taipei City Hospital (MK, DC); Institute of Public Health and Community Medicine Research Center, National Yang-Ming University (MK, YL, PC, DC); Department of Health Care Management, National Taipei University of Nursing and Health Sciences (MK, CC, DC); Department of Dentistry, Taipei City Hospital (YL); and Department of Dentistry, School of Dentistry, National Yang-Ming University, Taipei, Taiwan (YL).

## Abstract

Frequent emergency department (ED) users contribute to a disproportionate number of ED visits that consume a substantial amount of medical resources. Additionally, people with frequent ED visits may be at greater risks of illnesses and injury and are vulnerable to even more severe health events. We conducted, based on a nationally representative sample, a population-based study to estimate the prevalence of frequent ED users among all ED users, and to explore factors associated with frequent ED visits.

This is a population-based cross-sectional study. Data of 1 million people randomly selected from all beneficiaries of Taiwan's National Health Insurance claim database in 2010 were analyzed to estimate the distribution of ED visit among ED users. Multivariate logistic regression was employed to calculate the independent associations of factors with prevalence of frequent (4-12 ED visits per year) and highly frequent (>12 ED visits per year) ED visits.

Of the 1 million beneficiaries 170,475 subjects used ED service in 2010 and 103,111 (60.5%), 37,964 (22.3%), 14,881 (8.7%), 14,041 (8.2%), and 460 (0.3%) subjects had 1, 2, 3, 4 to 12, and more than 12 ED visits, respectively. ED users with 4 to 12 visits and those with >12 visits disproportionally accounted for 24.1% and 3.0%, respectively, of all ED visits in 2010. We noted significant associations of frequent ED visit with a number of factors including socio-demographics, health care utilization, and comorbidity. Among them, the most increased adjusted odds ratio (AOR) was noted for hospitalization during the past year (AOR = 1.85) and younger ages (1–6 years) (AOR = 1.84). On the contrary, the significant predictors for highly frequent ED visit with greater AOR included hospitalization during the past year (AOR = 3.95), >12 outpatient visits during the past year (AOR = 2.66), and a history of congestive heart failure (AOR = 2.64) and psychiatric disorders (AOR = 2.35).

People admitted and with frequent outpatient visits were at greater risk of frequent ED visit. Because people with a history of various comorbidities were also vulnerable to become frequent ED users, careful management of those comorbidities by clinicians may help further reduce the likelihood of frequent ED visit.

## INTRODUCTION

The demand for emergency care is increasing. The UK, New Zealand, Canada, and the USA report increased emergency department (ED) attendance, with current rates ranging from 3% to 6% annually.^1^ Over the past decade, the increase in ED utilization has outpaced growth of the general population, despite a national decline in the total number of ED facilities.^2,3^

In hospitals with insufficient inpatient bed availability, the increase in ED visits and in the number of emergency patients who require admission may increase length of stay in the ED, leading to overcrowding and compromised ED performance.^4,5^ These issues also result in the increased burden of caring for patients awaiting admission, as well as prolonged waiting times at the ED, more patients leaving without being seen, and ED blockage.^6^ Such disruptions of timely ED care may pose a threat to patient safety.^5,7^

As hospital EDs have experienced a dramatic increase in patient volume, interest has focused on the groups of individuals who contribute a disproportionate number of visits. Previous studies reported that frequent ED users composed 4.5% to 8% of all ED patients but accounted for 21% to 28% of all ED visits.^8^ Hunt et al^9^ reported that 92% of adult users made 1 to 3 visits, accounting for 72% of all adult ED visits, and that the 8% of users with ≧4 visits were responsible for the remaining 28% of adult ED visits from July 2000 through June 2001.

Previous studies on frequent ED use have raised doubts about commonly held assumptions. Frequent ED users were more likely than less-frequent ED users to be poor or near-poor.^10^ Seventy percent of frequent users were homeless or qualified for public assistance, and they often visited EDs for shelter, safety, rest, food, clothing, and social interaction.^11^ Frequent ED users were more likely to use other health care services.^12^ In addition, frequent ED users were more likely than less-frequent ED users to have more outpatient visits to physicians and a perception of unmet medical needs.^10^ The subjects in poorer health were more likely to be frequent ED users.^9^

However, much remains unclear about frequent ED users. For example, there is no widely accepted definition of a frequent ED user, and the definitions of frequent use range from as few as 3 visits annually to 12 or more visits annually, often without a clear rationale for the designation.^10,13–16^ Very few studies^9^ have presented the distribution of the number of ED visits in their studied population to define a cutoff for frequent ED use and to provide a rationale for their definitions of frequent use. Thus, it is difficult to compare or integrate the results of these studies. Most of the previous studies on frequent ED use were hospital based, which makes the results difficult to generalize. Moreover, frequent ED users may visit multiple EDs. One study reported that 58% of frequent users in Massachusetts visited 2 or more EDs in a 12-month period.^17^ Some of the population-based studies were based on either self-reported ED use of uncertain accuracy^9,10,18^ or large administrative databases that have limited information on important patient characteristics.^19^

The national health insurance (NHI) program in Taiwan is a universal health insurance program that was implemented in 1995.^20^ Data from the NHI provide detailed information on ED users, including the diagnoses of their physical illnesses and psychiatric disorders (PDs), records of outpatient health service visits and hospitalization, and the welfare status of the beneficiaries. In this study, nationally representative data from the NHI were used to describe the distribution of ED visit frequency among ED users and to characterize frequent ED users.

## METHODS

This study aimed to describe the distribution of the frequency of ED visits among ED users in 2010 and to evaluate the association of frequent ED use with various patient characteristics, including age, gender, whether the patient was receiving social welfare, utilization of other health care resources, including outpatient visits and hospitalization in the previous 1 year, and comorbidities.

### Study Design and Data Source

This is a population-based cross-sectional study. The data were obtained from the National Health Insurance Research Database (NHIRD), a large-scale computerized database supervised by the Health Promotion Administration, Ministry of Health and Welfare, and maintained by the National Health Research Institutes (NHRI). NHIRD is provided to local scientists in Taiwan for research purposes. Data of NHIRD that can be used to identify patients or care providers, including medical institutions and physicians, are scrambled before it is sent to the NHRI for database construction. Data are further scrambled before it is released to each researcher. Therefore, individual patient or health care providers cannot be identified from the database.^21^

The NHI program has enrolled approximately 99% of the Taiwanese population, and the Bureau of NHI had contracted with 97% of the hospitals and clinics throughout the nation by the end of 1996.^20^ After approval from institution review board of Taipei City Hospital and ethical approval from the NHRI, all of the ambulatory care claims (years 2009–2010), all of the in-patient claims (years 2009–2010), and the updated registry for beneficiaries (year 2010) of 1 million subjects randomly selected from all of the beneficiaries in 2010 were used in this study. According to the NHI database, no significant differences in the age or sex distributions existed between the beneficiaries in the 1-million-subject sample and the original population of all of the beneficiaries.^22^ The ambulatory care expenditure by visit (ACEV) files provide information on date of visit, up to 3 diagnoses, scrambled identification numbers of both the patients and the attending physicians, patient sex, and date of birth. In addition, the ACEV files provide codes for the physician fees for emergency care, which can be used to identify ED visits. Using the scrambled individual personal identification number, we were able to link all of the datasets.

### Selection of the Study Participants and Outcome Measurements

All of the ED visits in 2010 were analyzed to calculate the number of ED visits for each individual. The age of each study subject was calculated by the difference in time between the index date and the date of birth. The status of receiving welfare was identified from an updated registry of the beneficiaries. The numbers of outpatient visits and hospitalizations in 1 year prior to the first ED visit were calculated. We evaluated the individuals’ comorbidities, including PDs and the diseases included in the Charlson comorbidity index, which considers 19 predetermined clinical conditions and is a strong predictor of various adverse clinical outcomes.^23^ We searched the ACEV files for 2009 to 2010 and counted those comorbidities only when the subjects had at least 3 out-patient visits with the diagnosis 1 year prior to the first ED visit.

### Statistical Analysis

Descriptive statistical analysis was used to illustrate the distribution of the frequency of ED visits among ED users. We divided the ED users into nonfrequent ED users, frequent ED users, or highly frequent ED users after considering the characteristics of the different levels of the frequent ED users. To investigate the independent effects of various patient characteristics, patient utilization of other health care resources, and the comorbidities on frequent ED use, we used multivariate logistic regression. All statistical analyses were performed using SAS statistical software (version 9.1; SAS institute, Cary, NC). A *P* value <0.05 was considered statistically significant.

## RESULTS

**FIGURE 1:**
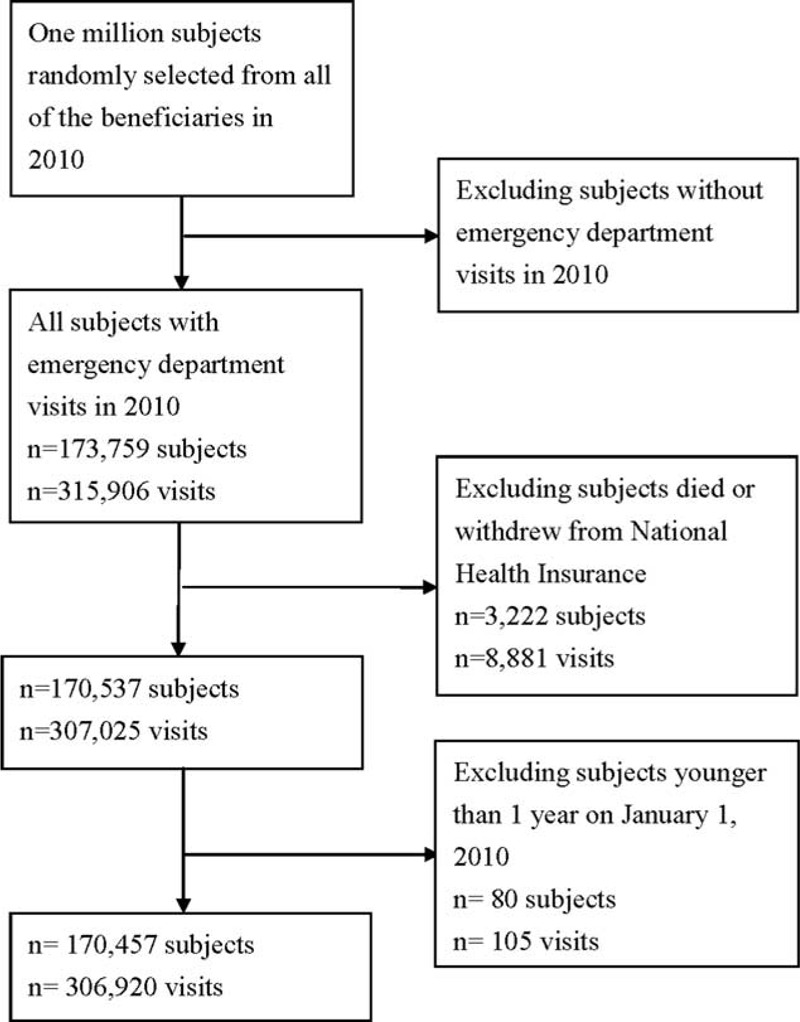
The process of selection of subjects with emergency department visits in year 2010.

The process of selecting the subjects with ED visits in 2010 is shown in Figure 1. Of the 1 million beneficiaries 173,759 subjects used ED service in 2010. After excluding subjects who died or withdrew from the NHI program in 2010 and those aged <1 year on January 1, 2010, there were 170,457 subjects, accounting for 306,920 ED visits in 2010 (Figure 1). We excluded those aged <1 year because we defined comorbidities as having at least 3 out-patient visits with the diagnosis 1 year prior to the first ED visit.

**FIGURE 2:**
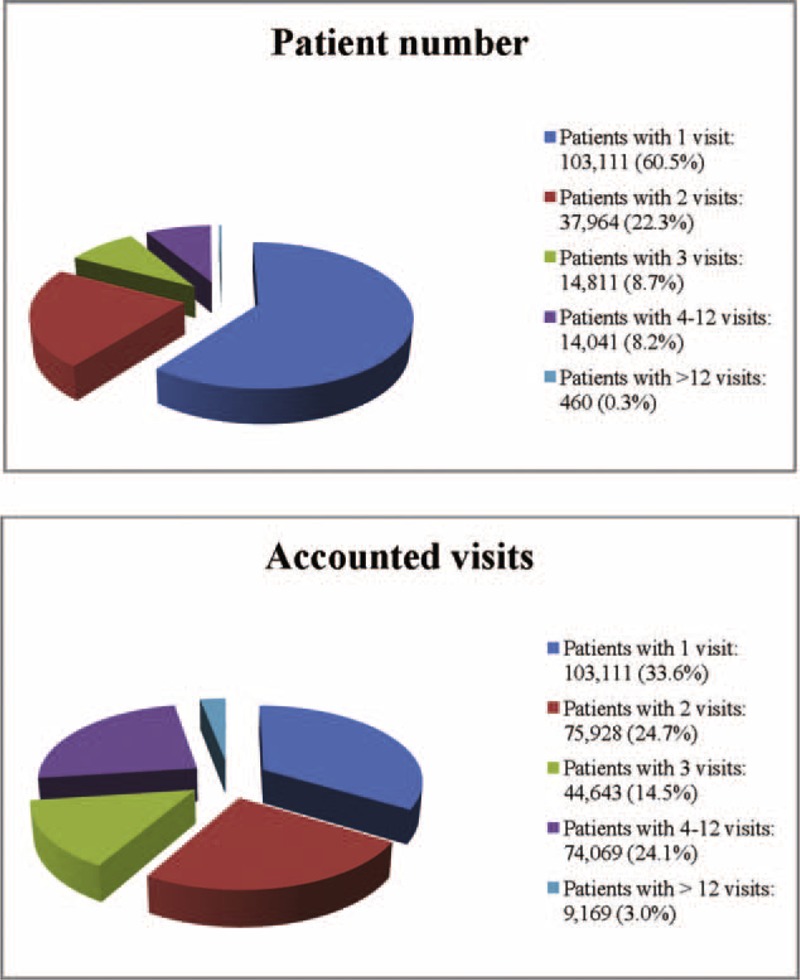
Distribution of frequency of emergency department visits among emergency department users.

The number of subjects with 1, 2, 3, 4 to 12, or >12 ED visits was 103, 111 (60.5%), 37,964 (22.3%), 14,881 (8.7%), 14,041 (8.2%), and 460 (0.3%), respectively. Among the ED users, 91.5% of the subjects visited the ED 1 to 3 times, and they accounted for 72.9% of the ED visits in 2010. Those with 4 to 12 ED visits and >12 ED visits accounted for 24.1% and 3.0%, respectively, of all the ED visits (Figure 2). According to the distribution of the frequency of ED visits shown in our study and in the study by Hunt et al in 2006,^9^ we defined the subjects with 1 to 3 ED visits as nonfrequent ED users, those with 4 to 12 ED visits as frequent ED users and those with >12 ED visits as highly frequent ED users.

Compared with subjects aged 18 to 44 years, those aged 1 to 6 years had a higher rate of frequent ED users (12.67%), and those aged ≧65 years had higher rates of frequent ED users (15.34%) and highly frequent ED users (0.57%). A higher percentage of male were frequent ED users (8.5%) or highly frequent ED users (0.29%). People receiving welfare had a higher rate of being frequent ED users (11.39%) or highly frequent ED users (0.52%). Subjects with >12 outpatient visits in the previous 1 year were more likely to be either frequent ED users (10.66%) or highly frequent ED users (0.40%). Subjects who had been hospitalized in the previous 1 year had a greater likelihood of being either frequent ED users (15.43%) or highly frequent ED users (0.84%) (Table 1). With regard to comorbidities, subjects with diseases included in the Charlson comorbidity index, except for subjects with acquired immune-deficiency syndrome, were more likely to be frequent ED users (9.09%–22.48%) or highly frequent ED users (0.59%–2.02%). Subjects with PDs were more likely to be frequent ED users (15.22%) or highly frequent ED users (1.05%) (Table 2).

**TABLE 1:**
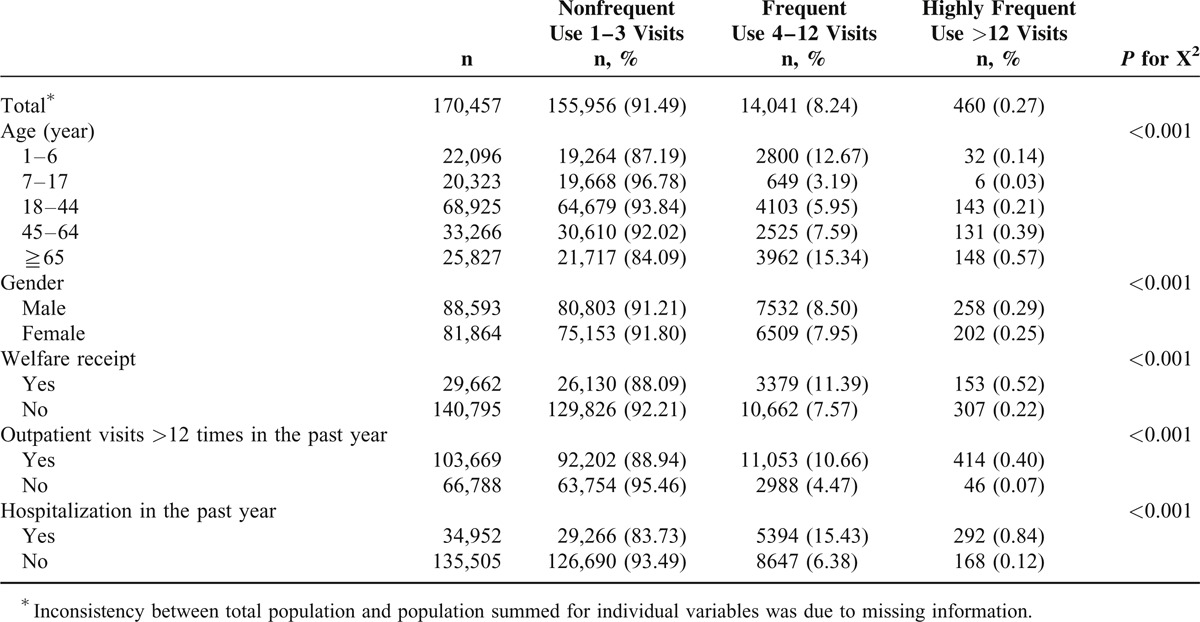
Frequency of Emergency Department Visits According to Patient Characteristics, and Utilization of Outpatient/Inpatient Utilization

**TABLE 2:**
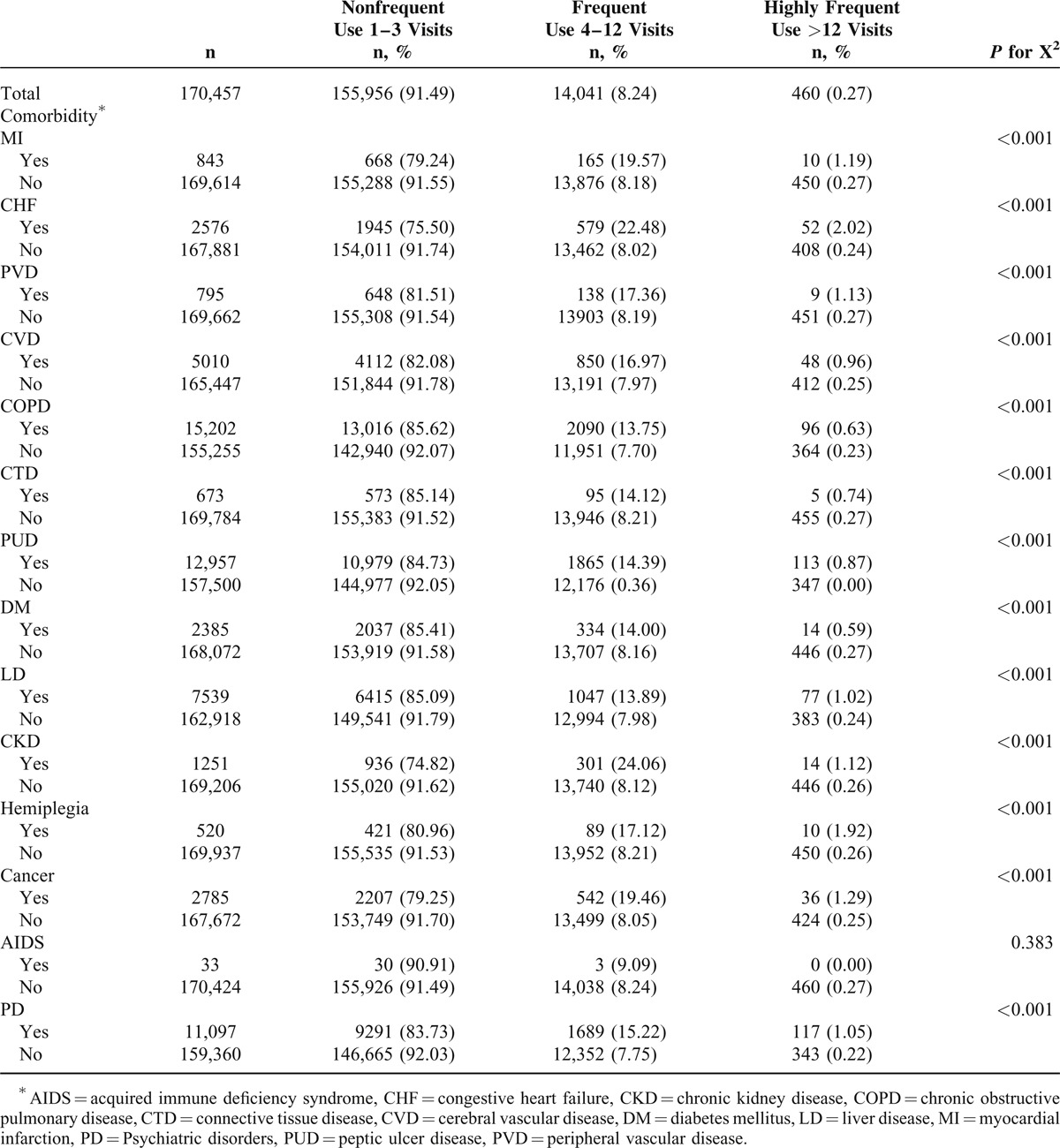
Frequency of Emergency Department Visits According to Comorbidities

Table 3 demonstrates the independent association of frequent ED use or highly frequent ED use with various patient characteristics. Compared with subjects aged 18 to 44 years, those aged 1 to 6 years (adjusted odds ratio [AOR]: 1.84) and those aged ≧65 years (AOR: 1.77) had a significantly higher risk of being frequent ED users. In contrast, subjects aged 7 to 17 years were less likely to be frequent ED users (AOR: 0.54) or highly frequent ED users (AOR: 0.17). Male gender (AOR = 1.16 and 1.43, respectively) and receiving welfare (AOR = 1.37 and 1.81, respectively) increased the risk of both frequent ED use and highly frequent ED use. More than 12 outpatient visits (AOR = 1.59 and 2.66, respectively) and hospitalization (AOR = 1.85 and 3.95, respectively) in the previous 1 year were associated with frequent ED use or highly frequent ED use. With regard to comorbidities, congestive heart failure (CHF, AOR = 1.39), peripheral vascular disease (AOR = 1.24), cerebral vascular disease (AOR = 1.12), chronic obstructive pulmonary disease (COPD, AOR = 1.24), connective tissue disease (AOR = 1.32), peptic ulcer disease (PUD, AOR = 1.34), liver disease (LD, AOR = 1.30), chronic kidney disease (AOR = 1.61), cancer (AOR = 1.34), and PD (AOR = 1.57) increased the risk of frequent ED use. CHF (AOR = 2.64), COPD (AOR = 1.40), PUD (AOR = 1.57), LD (AOR = 1.85), cancer (AOR = 1.47, 95% CI: 1.03–2.10), and PD (AOR = 2.35) increased the risk of highly frequent ED use (Table 2).

**TABLE 3:**
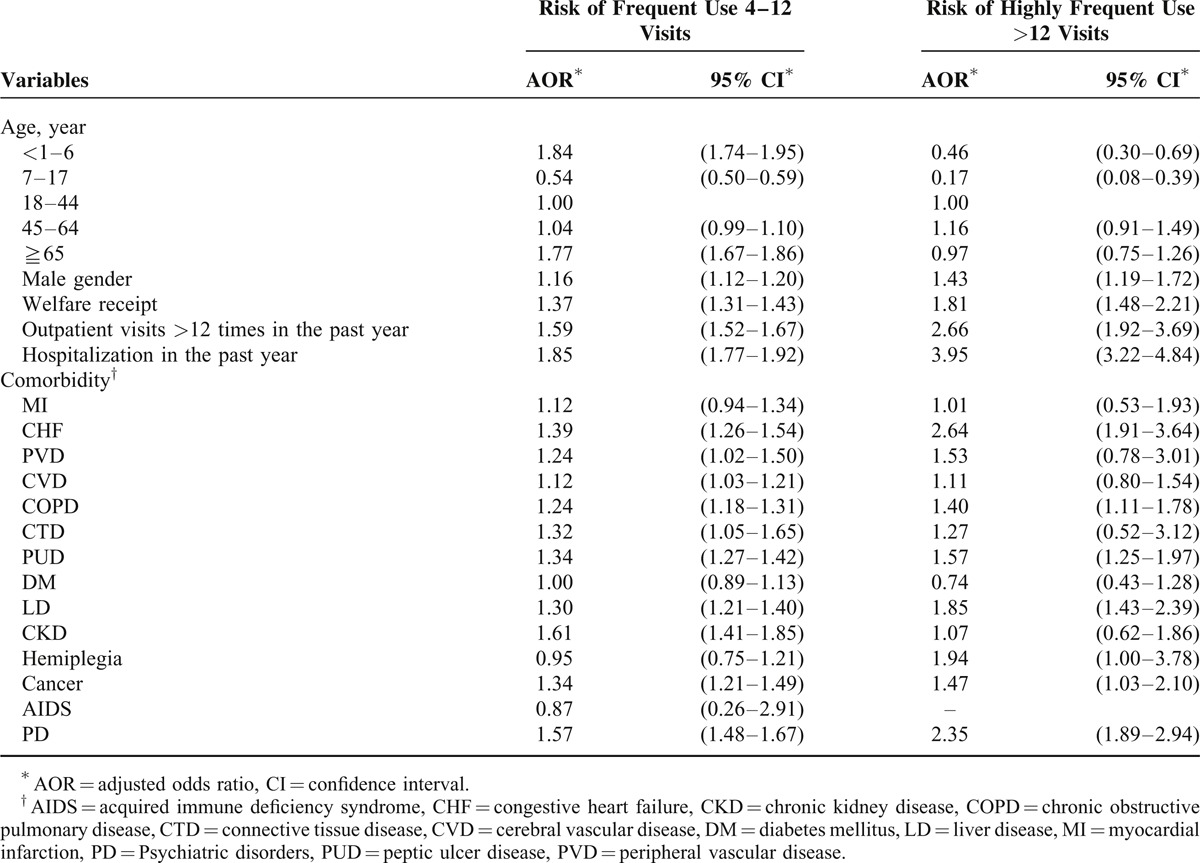
Associations of Frequency of Emergency Department Visits with Patient Characteristics, Outpatient/Inpatient Utilization, and Comorbidities

## DISCUSSION

In our study, ED users with 4 to 12 visits and those with >12 visits disproportionally accounted for 24.1% and 3.0%, respectively, of all ED visits. We noted significant associations of frequent ED visit with a number of factors including socio-demographics, health care utilization, and comorbidity. Among them, the most increased AOR was noted for hospitalization during the past year and younger ages (1–6 years). On the contrary, the significant predictors for highly frequent ED visit with greater AOR included hospitalization during the past year, >12 outpatient visits during the past year, and a history of CHF and PD.

The definitions of frequent ED use in previous studies were inconsistent, with a range from 3 visits annually to 12 or more visits annually, without a clear rationale for the chosen designations.^9^ Zuckerman and Shen^10^ defined frequent ED use as 3 or more ED visits annually, with the rationale that any individual might have a small number of ED visits but that “having 3 or more ED visits might reflect dependence on the ED as a source of care.” Chan and Ovens^16^ used 12 ED visits based on identifying outliers in health care seeking behavior and the ability of ED physicians to “recognize 1 visit per month.” Although the authors provided the reasons for the definition of frequent ED use, the distribution of the visits was not demonstrated and the number of visits accounted for by frequent ED users was not reported. Very few studies^9^ have demonstrated the overall distribution of ED visits in their study population to define a cutoff for frequent ED use. In the study by Hunt et al, the authors utilized a population-based, nationally representative Community Tracking Study Household Survey to identify the characteristics of frequent ED users. They calculated the number of adults (aged 18 and older) who visited the ED 1 to 7 or more times and the number of associated visits. Based on the distribution of visits, they established a definition for a frequent user of 4 or more visits. They reported that 92% of the adult users had 3 or fewer visits, accounting for 72% of all the adult ED visits; the 8% of users with 4 or more visits were responsible for the remaining 28% of adult ED visits.^9^ In our study, we analyzed the frequency of ED visits among ED users and divided the ED users into nonfrequent ED users (1–3 visits), frequent ED users (4–12 visits), and highly frequent ED users (>12 visits), as it was likely that the characteristics of the frequent and highly frequent users might differ. Our results revealed that the frequent ED users and highly frequent ED users contributed a disproportionate number of ED visits. Furthermore, differences between the frequent ED users and the highly frequent ED users were also identified.

With regarding the association between age and frequent ED use, Milbrett and Halm^24^ reported that age 30 to 54 years was one of the common characteristics of frequent ED users (≧6 ED visits annually). A statewide analysis of ED utilization in Massachusetts reported that higher proportions of people aged 25 to 44 or >65 were frequent ED users (≧5 ED visits annually).^17^ One study on the utilization of ED care in Taiwan reported that subjects aged 0 to 9 years accounted for the highest proportion (19.2%) of ED visits in 2004 and that those aged 10 to 19, 20 to 29, 30 to 39, 40 to 49, 50 to 59, and ≧60 years accounted for 9.7%, 18.0%, 13.0%, 11.9%, 9.2%, and 19.0%, respectively, of ED visits.^25^ In our study, those aged ≧65 years were more likely to be frequent ED users than those aged 18 to 44 years. Elderly people were more likely to have chronic diseases and multiple comorbidities,^26^ which may increase the likelihood of ED use.^27^ In our study, children aged 1 to 6 years had a higher risk of frequent ED use, while those aged 7 to 17 years had a lower risk of frequent ED use. The study by LeDuc on pediatric ED recidivism, in a tertiary care, academic children's hospital reported that compared with those aged <1 year, the odds ratio of return to the ED within 3 months for those aged 1 to 4, 5 to 12, and ≧13, was 0.47, 0.47, and 0.39, respectively.^28^ In addition a principle diagnosis that falls under the broad category of nervous system and sense organ diseases have a much higher chance of returning to the ED.^28^ One study on characteristics of frequent pediatric ED users (≧10 ED visits annually) reported that among 357 subjects aged <21 years, 265 (74%) had chronic disease conditions. The most common chronic medical conditions were recurrent wheezing, followed by neurologic conditions, gastrointestinal conditions, cardiac conditions, and endocrine conditions.^29^

With regard to the association of patient gender with frequent ED use, previous studies have reported inconsistent results. One descriptive study at the ED of an academic hospital reported that the sex distribution of frequent ED users with more than 12 visits was similar to that of general ED patients.^13^ Another study at the ED of a teaching hospital in London (years 2006–2007) reported that frequent visitors were more likely to be men than women (50.5% of single visits; 69.5% of ≧10 visits).^30^ By contrast, One study in Massachusetts reported that females represented a higher proportion of frequent ED users (≧5 visits) than males.^17^ Our study demonstrated that male gender increased the risk of both frequent ED use and highly frequent ED use. One study on the ecology of medical care in Taiwan reported that the overall monthly ED utilization rate was 18.9/1000 and a higher proportion of men than women (9.8/495.8 versus 9.0/504.2) received emergency services in 2005.^31^ One study in Hong Kong reported that the monthly ED utilization rate was higher in male than in female (17.2/1000 versus 15.5/1000).^32^ Another study in Sweden reported that female had a significantly lower risk for ED utilization (AOR = 0.94, 95% CI: 0.92–0.96).^33^ Other studies have argued that women are possibly more health conscious^34^ and that men usually seek medical help at a later stage of illness.^35^ In addition, women may have lower employment rates^36^ and thus more time to visit physicians during office hours. In addition to health-seeking behaviors and custom between genders, culture in different areas might also affect the sex difference in ED utilization.

In our study, receiving welfare increased the risk of both frequent ED use and highly frequent ED use. This result was consistent with those of some previous studies. The study by Zuckerman and Shen^10^ found that frequent ED users were more likely than less-frequent ED users to be poor or near-poor. One study in a universal health insurance system in Switzerland reported that being unemployed or dependent on government welfare increased the risk of frequent ED use.^37^ Hunt et al^9^ reported that a family income below the poverty threshold increased the risk of frequent ED use.

Similar to previous studies, our study demonstrated that more than 12 outpatient visits in the previous 1 year significantly increased the risks of frequent ED use and highly frequent ED use.

Interestingly, both LaCalle and Rabin^8^ and Hunt et al^9^ reported that frequent ED users were also heavy users of other sectors of the health care system. Hunt et al^9^ further indicated that these patients were more likely to be dissatisfied with their medical care and that most adults who frequently used the ED had a usual source of care.^9^ Additionally, Bieler et al^37^ reported that the use of 5 or more clinical departments over 12 months increased the risk of frequent ED use.

Consistent with previous studies, our study demonstrated that hospitalization in the previous 1 year increased the risk of frequent ED use and highly frequent ED use. McCusker et al^38^ reported that recent hospitalization, an indicator of the severity of an illness, was an important predictor of early and frequent ED returns. Sun et al^39^ reported that hospitalization in the preceding 3 months predicted frequent ED use.

In our study, comorbidities, including CHF, COPD, PUD, LD, and cancer, significantly increased the risks of frequent ED use and highly frequent ED use. There is a marked heterogeneity in the predominant types of complaints reported by frequent ED users. Some studies have reported a preponderance of exacerbations of chronic illnesses (eg, renal failure, COPD/asthma, and sickle cell disease),^10,40–43^ whereas others describe many visits that are attributable to less-specific symptomatology and pain.^13,44^ Using the national data from the Veterans Health Administration in the USA, Doran et al^45^ reported that heart failure was strongly associated with all levels of ED use. Additionally, McCusker et al^38^ reported that patients with a history of heart disease and patients with digestive diagnoses were more likely to return at an earlier date to the ED and that patients with respiratory diagnoses were more likely to return frequently.

In our study, PD significantly raised the risks of frequent ED use and highly frequent ED use. This result is consistent with those of previous studies, as Bieler et al^37^ reported that psychiatric hospitalization increased the risk of frequent ED use while Hunt et al^9^ indicated that poor mental health increased the risk of frequent ED use. Furthermore, Sandoval et al^46^ stated that frequent ED visitors were much more likely to screen positively for depression. Sun et al^39^ reported that a high rating of psychological distress was a predictor of frequent ED use. Indeed, government policies across the developed world have encouraged the mainstreaming of care from long-stay psychiatric hospitals to community-based settings and such policies are thought to have contributed to increased ED visits by patients with mental health problems.^1^ One study in Australia reported a 10-fold increase in the number of patients attending the ED with primarily mental health problems during the 10 years from 1993/94 and the percentage of mental health patients has risen from 0.3% to more than 3.5%.^47^ Studies in Australia, the UK, and Europe also reported high incidences of psychiatric illnesses and substance abuse among repeat ED visitors in the last decade.^48–50^

Frequent ED users contribute substantially to ED crowding, and there is a concern that their use of EDs might be inappropriate. Some previous studies reported that frequent ED users were more likely to present with primary care complaints that were better treated elsewhere.^51,52^ However, our study revealed that the risks of frequent ED use and highly frequent ED use were increased by more than 12 outpatient visits or hospitalization in the previous 1 year, PDs and diseases included in the Charlson comorbidity index, in addition to receiving welfare. The results of our study suggest that frequent ED users have greater health care needs and may be using the ED appropriately or perhaps in lieu of other forms of care that are unavailable to them.

Frequent utilization of the ED is a challenging and contentious issue for clinicians and policy-makers. A number of interventions, including case management, individualized care plans, and information sharing, aimed at reducing the number of ED visits by frequent users have been evaluated in the literature.^53^ In response to the growing challenges of some devastating disease, the National Health Insurance Administration in Taiwan has initiated a multidisciplinary care program since 2001 aiming at improving care.^54^ Further studies could be conducted to evaluate the impact of multidisciplinary care program on ED utilization.

Our study has several methodological strengths. First, using insurance claims data in clinical research allows easy access to the longitudinal records of a large sample of geographically dispersed patients and greatly increased the representativeness of the study sample. Second, the NHI dataset provided additional details and more accurate information on the ED users, which reduced the recall bias. Several study limitations also warrant discussion. First, because we used the linked administrative data, we had little information on the clinical presentations during the ED visits, which may have confounded the study results. Second, some socioeconomic data were not available, and such data could be vital for describing the characteristics of ED users. Third, some ED visitors may not claim their ED visits or choose to claim with other health insurance programs although the proportion of ED visits without NHI claim should be small under a universal NHI system.

## CONCLUSIONS

In our study, frequent ED use was associated with the older age group, male gender, and welfare status, more than 12 outpatient visits or hospitalization in the previous 1 year, PDs and diseases included in the Charlson comorbidity index. The results of our study suggest that frequent ED users may visit the ED appropriately. A comprehensive policy including a diversionary strategy for the different causes of frequent ED use may be needed to reduce the ED utilization by frequent ED users.
